# PTEN is involved in modulation of vasculogenesis in early chick embryos

**DOI:** 10.1242/bio.20133988

**Published:** 2013-05-09

**Authors:** Yan Li, Xiao-yu Wang, Ting Wu, Manli Chuai, Kenneth Ka Ho Lee, Li-jing Wang, Xuesong Yang

**Affiliations:** 1 Key Laboratory for Regenerative Medicine of The Ministry of Education, Department of Histology and Embryology, School of Medicine, Jinan University, Guangzhou 510632, China; 2 Institute of Vascular Biological Sciences, Guangdong Pharmaceutical University, Guangzhou 510006, China; 3 Division of Cell and Developmental Biology, University of Dundee, Dundee DD1 5EH, UK; 4 Stem Cell and Regeneration Thematic Research Programme, School of Biomedical Sciences, Chinese University of Hong Kong, Shatin, Hong Kong

**Keywords:** Chick embryo, *PTEN*, Vasculogenesis, Blood islands, Cell migration

## Abstract

*PTEN* is a tumor suppressor gene that is mutated and/or deleted in many types of tumor. This gene also plays a very distinct role in the early stages of embryonic development such as cell migration, proliferation and migration. Nevertheless, little is known of the function of *PTEN* in vasculogenesis during chick embryonic development. In this study, we used *in situ* hybridization to first demonstrate the expression pattern of *PTEN* during gastrulation. *PTEN* was found mainly expressed in the blood islands of area opaca, the neural tube and mesodermal structures. Overexpression of *PTEN* obstructed the epithelial–mesenchymal transition (EMT) process in the primitive streak. EMT is the first prerequisite required for the emigration of hemangioblasts during vasculogenesis. When *PTEN* expression was silenced, we observed that it produced an adverse effect on mesodermal cell emigration to the extra-embryonic blood islands. In addition, we also demonstrated that even if the perturbed-*PTEN* cells did not affect the formation of blood islands, migrant mesodermal cells overexpressing wt PTEN-GFP had difficulties integrating into the blood islands. Instead, these cells were either localized on the periphery of the blood islands or induced to differentiate into endothelial cells if they managed to integrate into blood islands. Development of the intra-embryonic primary vascular plexus was also affected by overexpression of *PTEN.* We proposed that it was elevated *PTEN* lipid phosphatase activity that was responsible for the morphogenetic defects induced by *PTEN* overexpression. In this context, we did not find *PTEN* affecting *VEGF* signaling. In sum, our study has provided evidence that *PTEN* is involved in vasculogenesis during the early stages of chick embryo development.

## Introduction

In the developing chick embryo, vasculogenesis involves the differentiation of angioblasts from mesodermal cells and the formation of primary capillary plexuses from angioblasts (Risau and Flamme, 1995). Vasculogenesis takes place in the blood islands of area opaca located in the yolk sac. The blood islands harbor not only angioblasts but also hematopoietic cells (Dieterlen-Lievre et al., 1988). Hemangioblasts are the common precursor cells of both angioblasts and hematopoietic cells. Vasculogenesis has been considered as being different from angiogenesis because of the different origins of the endothelial progenitor cells. For vasculogenesis, the endothelial progenitor cells are derived directly from mesodermal cells whereas in angiogenesis the endothelial progenitor cells are derived from the primary capillary plexuses. Moreover, vasculogenesis is generally considered an embryonic event whereas angiogenesis is regarded as a process that takes place in the adult. It appears now that the concept of vasculogenesis and angiogenesis as being different processes may not be accurate (Eichmann et al., 2002; Drake, 2003; Kässmeyer et al., 2009). In this context, we revisited the developmental events associated with vasculogenesis in the developing chick embryo.

During gastrulation, the mesodermal cells migrate out of the primitive steak and aggregate and assemble into blood islands. The soluble growth factor, VEGF, is expressed in the blood islands and appears to play a crucial role in vascular development (Koibuchi et al., 2006). VEGFR2 and several transcription factors, GATA-1, -2, SCL/tal-1 and Lmo2, have been demonstrated to be indispensable modulators of hematopoietic cells and commitment to the endothelial cells fate (Minko et al., 2003). It has been reported that VEGFR2 is crucial for maintaining endothelial cells development and that homozygous VEGFR2 mutants were not viable. These mutants die round E8–E9.5 due to improper development in hematopoietic and endothelial cells. In VEGFR2 knockout mice, the blood islands are barely visible in the yolk-sac and also inside embryo – suggesting a pivotal role for VEGFR2 in vasculogenesis (Flamme et al., 1995; Shalaby et al., 1995). In addition to VEGF, fibroblast growth factor (FGF) has also been identified as an inducer of blood islands development (Yasuda et al., 1992; Poole et al., 2001; Murakami and Simons, 2008). *In vitro* experiments demonstrated that FGF rather than TGF or EGF induced the endothelial cells (derived from the epiblasts) to aggregate into a characteristic vascular structure (Flamme and Risau, 1992). During blood islands formation, a proper cell–cell adhesion is also important for maintaining the integrity of the primary vascular plexus formed by the migrant mesodermal cells. This cell–cell interaction is determined by adhesion molecules, PECAM and VE-Cadherin, expressed by cells located on the lateral borders of the early chick embryo (Risau and Flamme, 1995).

*PTEN* (phosphatase and tensin homolog) is a candidate tumor suppressor gene (Li et al., 1997; Steck et al., 1997). It has been reported that mutation of this gene is associated with many types of human tumors (Podsypanina et al., 1999; Birck et al., 2000; Zhou et al., 2002; Croushore et al., 2005; Stiles, 2009). In these tumors, *PTEN* is believed to be involved in the formation of blood vessels that supply the tumor cells. However, the blood vessels inside the tumors are morphologically different from vessels found in normal tissues. Besides differences in morphology, the tumor blood vessels are also dissimilar at the molecular and functional levels (Bussolati et al., 2010). Previously, we reported that *PTEN* is expressed in early chick embryo and play a pivotal role in guiding the emigration of mesodermal cell to their destinations during gastrulation (Leslie et al., 2007). Jiang et al. revealed that PI3K stimulated angiogenesis while overexpression of *PTEN* repressed the process in the yolk sac of developing chick embryos (Jiang et al., 2000). This implies that PI3K-AKT/PTEN signaling exerts a positive influence on embryonic angiogenesis (Jiang et al., 2000). Nevertheless, the exact role that *PTEN* plays in vasculogenesis, especially during the blood islands formation process, is still unclear.

In this study, we first proved that *PTEN* is endogenously expressed in the blood islands of chick embryonic yolk-sac. We then overexpressed *PTEN* to establish whether this would impair the emigration of mesodermal cells to blood islands and whether formation of intra-embryonic vascular plexus was affected. These findings were further validated by silencing *PTEN* expression in the gastrulating chick embryo. We demonstrated that overexpression of *PTEN* directed the mesodermal cells into the endothelial cell lineages and *PTEN* did not crosstalk with the VEGF signaling pathway.

## Materials and Methods

### Chick embryos

Fertilized leghorn eggs were acquired from the Avian Farm of South China Agriculture University. They were incubated in a humidified incubator (Yiheng Instruments, Shanghai, China) set at 38°C with 70% humidity. The eggs were incubated until the chick embryos reached the desired developmental stage (according to Hamburger and Hamilton, 1992; reprint of 1951 paper).

### Gene transfection and tissue transplantation experiment

HH2–3 (Hamburger and Hamilton stage 2–3) (Hamburger and Hamilton, 1992; reprint of 1951 paper) chick embryos were prepared for early chick culture, according to methods previously described (Chapman et al., 2001). The embryos were transfected with the *GFP* or *wt PTEN-GFP* gene by electroporation. Briefly, 0.5 µl plasmid DNA (1.5 mg/ml *GFP* or *wt PTEN-GFP*) was microinjected into the space between the vitelline membrane and the epiblast of chick embryos during gastrulation. The electroporation parameters used were as previously described (Yang et al., 2002). For one-sided gene transfection, the polarity of the pulses was kept constant. For electroporation on both sides of the embryo, the polarity of the electrodes was switched between pulses. After electroporation, the embryos were further incubated for 5 hours before the primitive streak tissues were used for transplantation. The labeled GFP*^+^* or wt PTEN-GFP*^+^* primitive-streak tissue was isolated from the posterior region of the streak and grafted into the same position and developmental stage of an untransfected host embryo. The embryos were then returned to the incubator for 30 hours, photographed and fixed for immunofluorescent staining and *in situ* hybridization.

### LY294002 administration

The LY294002 was added to EC culture medium with the concentration of 4 µM as previously described (Chapman et al., 2001). LY294002 was isolated at half side of the 35 mm culture dishes with a middle plastic barrier, and the another side as control. We put HH3 chick embryos to the home-made culture dishes. The embryo was put on culture dishes with anterior–posterior axis while primary streak underlying in the middle line. One side of embryo will be incubated in LY294002 culture medium, while another side of embryo is treated with DMSO as control. And then the embryos were incubated for 30 hours in a 38°C with 70% humidity incubator.

### Acetic carmine staining

Acetic carmine dye was prepared by adding 5 g carmine into 200 ml of 50% acetic acid. The solution was boiled in a water bath for 15 minutes and then filtered. Whole-mount chick embryos were exposed to the acetic carmine overnight, and then washed in distilled water for 10 minutes. Afterwards, the whole-mount embryos were destained in 1% hydrochloric acid in 70% ethanol until all of the embryonic structures could be seen in detail. The embryos were then transferred to glycerin until they were cleared.

### Immunofluorescent staining of whole-mount embryo

Immunofluorescent staining was performed on whole-mount embryo to reveal the presence of QH1, PTEN and AKT expression as previously described (Yang et al., 2008; Yue et al., 2008). Briefly, the embryos were fixed in 4% paraformaldehyde (PFA) at 4°C overnight, and unspecific immunoreactions were blocked using 2% Bovine Serum Albumin (BSA) + 1% Triton X-100 + 1% Tween 20 in PBS for 2 hours at room temperature. The embryos were then washed in PBS and incubated with primary monoclonal antibody mixture raised against QH1 (DSHB 1:100) or PTEN (6H2.1 Cascade BioScience 1:200) or AKT (Thr308 Cell Signaling 1:200) overnight at 4°C on shaker. After extensive washing, the embryos were incubated in specific secondary antibody conjugated to Alexa Fluor 488 dye (Alexa Fluor 555 goat anti-mouse IgG; Invitrogen, 1:1000) overnight at 4°C on a shaker to visualize the primary antibodies. After immunofluorescent staining, all the embryos were counterstained with DAPI (4′-6-Diamidino-2-phenylindole, Invitrogen, 5 µg/ml) for 1 hour at room temperature. Subsequently the embryos were sectioned on a cryostat microtome (Leica CM1900). The sections were mounted in mounting solution (Mowiol 4-88, Sigma) on glass slides and sealed with coverslips. All immunofluorescent staining were performed in replicates where at least 5–6 embryos were used.

### RNA-interference

A siRNA “smartpool” targeting the chicken *PTEN* gene was purchased from Dharmacon. The siRNA was diluted to a concentration of 1 mM in 20 mM KCl, 6 mM Hepes (pH 7.5) and 200 mM MgCl_2_. The 0.5 µl *PTEN-*siRNA was transfected into the chick embryos by microinjection and electroporation using methods described above. *In situ* hybridization was used to establish how extensively *PTEN* expression was silenced by *PTEN-*siRNAs.

### *In situ* hybridization

Whole-mount *in situ* hybridization of chick embryos was performed according to a standard *in situ* hybridization protocol (Henrique et al., 1995). Digoxigenin-labeled probes were synthesized against *PTEN* (Leslie et al., 2007), *VE-Cadherin* and *VEGFR2*. The whole-mount stained embryos were photographed and then frozen sections were prepared from them by cutting at thickness of 15–20 µm on a cryostat microtome (Leica CM1900).

### Photography

After immunofluorescent staining, the whole-mount embryos were photographed using stereoscope fluorescence microscope (Olympus MVX10) and imaging software (Image-Pro Plus 7.0). Sections of the embryos were photographed using an epi-fluorescent microscope (Olympus IX51, Leica DM 4000B) at 200 or 400× magnification using the Olympus software package Leica CW4000 FISH.

## Results

### PTEN expression in chick embryos during gastrulation

**Fig. 1.**
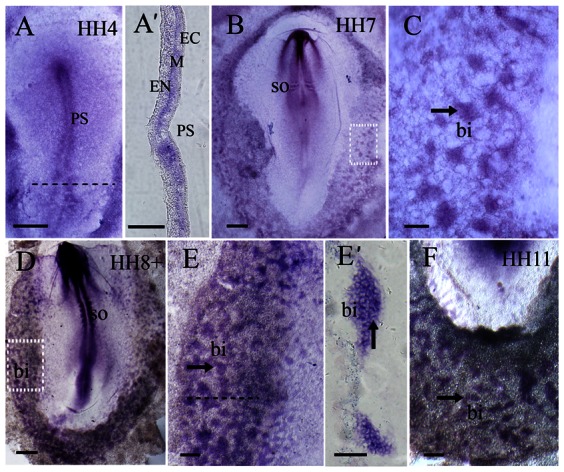
PTEN expression pattern during chick gastrulation. *PTEN* whole-mount *in situ* hybridization was performed on HH4, 7, 8 and 11 chick embryos. (**A**) In HH4 chick embryos, *PTEN* was expressed mainly in the primitive streak and the caudal boundary between area pellucida and area opaca – with the highest level of expression in the Hensen's node. (**A′**) Transverse section of the posterior primitive streak (level indicated by dotted line in A) revealed that *PTEN* was expressed in the ectoderm and lateral mesoderm. (**B**) In HH7 chick embryos, *PTEN* was mainly expressed in the head folds, neural plate and developing blood islands in the area opaca. (**C**) Higher magnification of the area opaca (dotted square outline in B) showed *PTEN* was expressed more prominently in the blood islands. (**D**) In HH8 embryo, *PTEN* was mainly expressed in primitive streak, neural tube, somites, presomitic mesoderm and blood islands of area opaca. (**E**) Higher magnification of the area opaca (dotted square outline in D) revealed *PTEN* was expressed more strongly in blood islands. (**E′**) Transverse section of the area opaca (level indicated by dotted line in E), showing *PTEN* expression was concentrated in the blood islands. (**F**) In HH11 embryo, *PTEN* was expressed in the blood islands of area opaca. Abbreviations: PS, primitive streak; EC, ectoderm; M, mesoderm; EN, endoderm; bi, blood islands; SO, somite. Scale bars: 500 µm in A,B,D; 100 µm in A′,C,E,F; 50 µm in E′.

*In situ* hybridization revealed that *PTEN* was first expressed in the Hensen's node and primitive streak of HH4 staged chick embryos (Fig. 1A). *PTEN* expression was strongest in the Hensen's node. During the primitive streak stage, *PTEN* is expressed on mesodermal cells, which will migrate laterally to the extra-embryonic area opaca. These *PTEN^+^* mesodermal cells could be observed in transverse sections of the posterior primitive streak (Fig. 1A′). In addition, another region of high *PTEN* expression was in the boundary area between the area opaca and pellucida at caudal end of the embryo (Fig. 1A). In HH7 chick embryos, *PTEN* was highly expressed in the head folds and the forming blood islands in extra-embryonic area opaca (Fig. 1B,C) – although *PTEN* expression within the blood islands was still weak. When embryos develop beyond HH8–HH11 stage, *PTEN* expression in the blood islands appeared much stronger (Fig. 1D–F) and it is particularly evident in transverse cryosections (Fig. 1E′). This spatiotemporal expression pattern for *PTEN* suggested that the gene might be involved in vasculogenesis during early embryonic development.

### Role of PTEN in hemangioblast migration from the primitive streak to the blood islands

It is now well established that the blood islands progenitor cells are derived mainly from the primitive steak. In order to determine whether *PTEN* played a role in hemangioblast migration, it was necessary to first confirm the hemangioblast migration trajectory from the posterior primitive streak to the blood islands-forming sites. This was achieved by transfecting a piece of the posterior primitive streak with the GFP marker and the transplanting it exactly into the same position and developmental staged (HH3) of a host chick embryo. Time-lapse recording of the first half of the cell migration trajectory demonstrated unequivocally that the migrating posterior primitive streak cells fanned out laterally and caudally to the area opaca (supplementary material Fig. S1).

We have established that *PTEN* was expressed at all developmental stages of vasculogenesis (Fig. 1). Consequently, we labeled the embryos with GFP at the primitive streak stage HH3 to investigate whether if *PTEN* played a role in mesodermal cell migration. GFP^+^ cells were found migrating laterally to the area opaca. These GFP^+^ cells were also found in the newly formed mesoderm germ layer. In contrast, overexpression of wt PTEN-GFP inhibited cell emigration from the primitive streak. It is consistent with observations by Leslie et al. for anterior streak cells inhibition of EMT (Leslie et al., 2007). A majority of the wt PTEN-GFP^+^ cells have accumulated in the primitive streak when examined 30 hours after transfection (supplementary material Fig. S2). These findings suggest that overexpressing *PTEN* inhibited the epithelial–mesenchymal transition (EMT) process in the primitive streak during chick gastrulation as shown in our supplementary data and our previous paper (Li et al., 2011).

### Effect of silencing PTEN on mesodermal cell number and blood islands formation

**Fig. 2.**
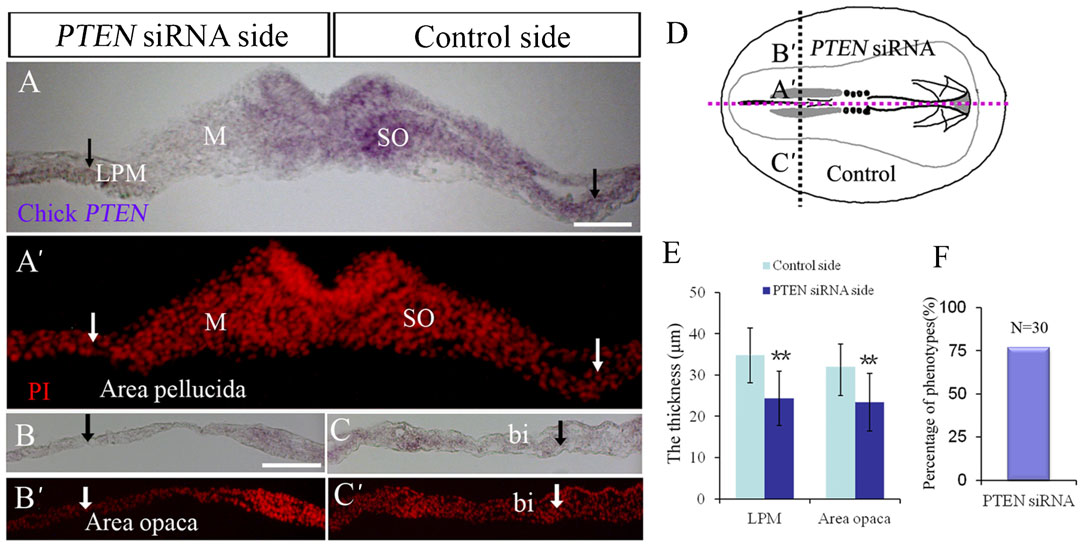
Silencing endogenous PTEN abridged the number of mesoderm cells. *PTEN* expression was silenced on half side of HH3–4 chick embryos using *PTEN*-siRNA. (**A**) *In situ* hybridization of embryo transverse sections confirmed that *PTEN* was silenced on the left side of the embryo following transfection. (**A′**) The thickness of the mesoderm on the silenced side was also thinner than the opposite control side in the area pellucida – suggesting that the mesoderm cell number was reduced by the *PTEN* silencing. (**B**,**B′**,**C**,**C′**) Transverse section of a representative embryo following *PTEN* silencing and control side in the area opaca. (B,C) *PTEN in situ* hybridization and PI staining (B′,C′). (**D**) The spatial relationship of B′ and C′ is shown in the schematically drawing. (**E**) The statistics of the thickness of lateral plate mesoderm and area opaca between *PTEN* siRNA sides and control side. (**F**) Showing the incidence of the percentage of phenotypes described. Abbreviations: M, mesoderm; SO, somite; bi, blood islands; LPM, lateral plate mesoderm. **P<0.01 vs control side. Scale bars: 100 µm in A–C′.

**Fig. 3.**
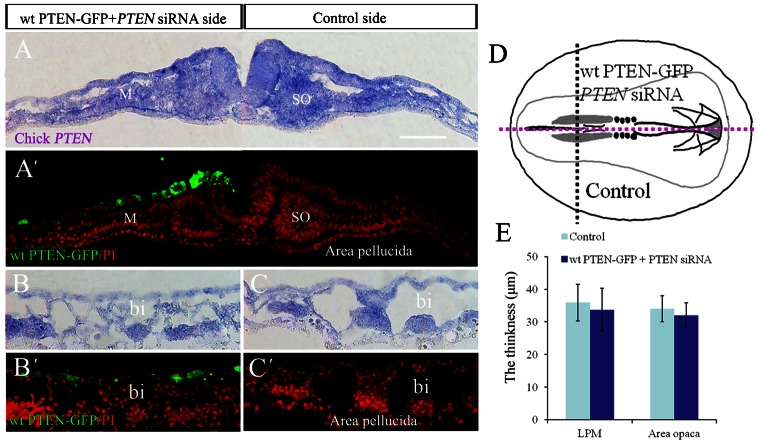
Co-transfection of *wt PTEN-GFP* and *PTEN* siRNA can rescue the adverse effect of downregulated PTEN, which induced the reduction of number of mesoderm cells and blood islands formation. (**A**) The transverse section of *PTEN* in situ hybridization demonstrates that the *PTEN* expression was rescued at left side of embryo. (**A′**) The cells were stained with PI in area pellucida, and the mesoderm cell number was not obviously changed by the *PTEN* siRNA and *wt PTEN-GFP* co-transfection between both sides. (**B**,**B′**) The transverse sections of PTEN in situ hybridization (B) and PI staining (B′) in PTEN co-transfection side of area opaca. (**C**,**C′**) The transverse sections of PTEN in situ hybridization (C) and PI staining (C′) in control side of area opaca. (**D**) The spatial relationship of B,B′ and C,C′ is shown in the schematically drawing. (**E**) The statistical data for the thickness of lateral plate mesoderm in both sides of embryos. Abbreviations: M, mesoderm; SO, somite; bi, blood islands; LPM, lateral plate mesoderm. Scale bar: 100 µm in A–C′.

We silenced *PTEN* expression on one side of the HH3 chick embryo using *PTEN*-siRNA to provide further evidence that the gene was involved in hemangioblasts migration from the primitive streak to the blood islands. We confirmed *PTEN* expression was silenced by *in situ* hybridization (Fig. 2A). The *PTEN*-siRNA transfected embryos were counterstained with propidium iodide to reveal the total number of cell present in the transverse sections (Fig. 2A′,B′). We established that there were fewer cells in the *PTEN* silenced side of the embryo (Fig. 2A′) than the opposite control side – which was obvious in both the lateral area pellucida and the area opaca (Fig. 2B,B′). In addition, the development of the blood islands was also repressed by the silencing of *PTEN* (Fig. 2B). The thickness of lateral plate mesoderm and area opaca was statistical between *PTEN* siRNA side and control side (Fig. 2E). The phenotype produced through silencing *PTEN* expression was observed in the vast majority of the transfected embryos (Fig. 2F). In addition, we also did the rescue experiment by co-transfection *PTEN* siRNA and *wt PTEN-GFP* (Fig. 3). Eventually, the thickness of lateral plate mesoderm in *PTEN* knockdown and overexpression co-transfection side was similar to one in control side as showed in Fig. 3E.

**Fig. 4.**
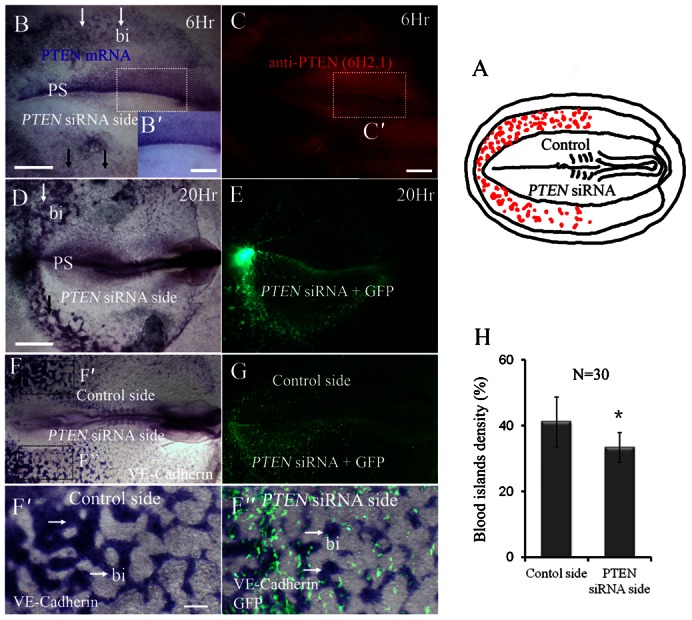
Silencing endogenous PTEN impairs blood islands formation in the area opaca. *PTEN* expression was unilaterally silenced in HH3–4 chick embryos using *PTEN*-siRNA as described schematically in panel **A**. (**B**,**B′**) *In situ* hybridizations confirming that *PTEN* has been unilaterally silenced, as indicated by the arrows, 6 hours after transfection. (**C**,**C′**) Immunohistological staining confirms that *PTEN* proteins have also been correspondingly reduced by *PTEN*-siRNA. (**D**) *In situ* hybridization for *PTEN*, 20 hours after transfection, showing in the *PTEN* silenced side area containing blood islands was significantly reduced (black arrows) as compared with the untransfected side of the embryo. (**E**) *GFP* and *PTEN*-siRNA co-transfection confirmed that the transfection worked, and that most of transfection disseminated in the lower half of the embryo. (**F**) In situ hybridization for *VE-Cadherin*, 20 hours after transfection, showing in the *PTEN* silenced side area containing blood islands was significantly reduced as compared with the untransfected side of the embryo. (**G**) *GFP* and *PTEN*-siRNA co-transfection confirmed that the transfection worked, and that most of transfection disseminated in the lower half of the embryo. (**F′**) *VE-Cadherin* labelled blood islands in the region of area opaca of control side. (**F″**) The merge image of GFP labelled co-transfection with *PTEN* siRNA and *VE-Cadherin* in situ hybridization, in which the blood islands labelled by *VE-Cadherin* presented sparser in comparison to the corresponding region of contralateral control side. (**H**) Chart showing the effect of blood islands density after transfection. Abbreviations: PS, primitive streak; bi, blood islands. *P<0.05 vs control side. Scale bars: 1 mm in B–G; 200 µm in B′,C′; 100 µm in F′,F″.

**Fig. 5.**
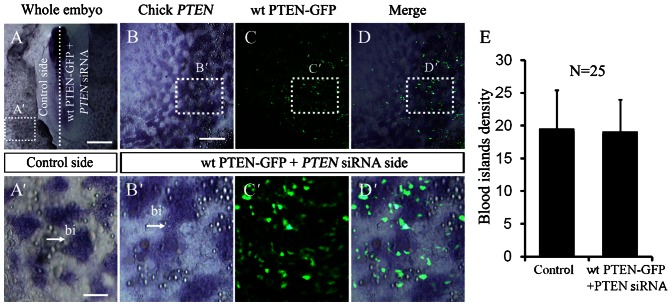
Simultaneously knocking down and overexpressing endogenous PTEN does not affect blood islands formation in chick area opaca. (**A**) Whole-mount *PTEN* in situ hybridization was performed to determine *PTEN* gene expression following simultaneously knocked down and overexpressed *PTEN* in chick embryo. (**A′**) The magnification image from the control side of area opaca indicated by dotted line square in panel A. (**B**) Chick *PTEN* expression in co-transfection side (right) of *PTEN* siRNA and *wt PTEN-GFP*. (**B′**) The magnification image from dotted line square in panel B. (**C**) *Wt PTEN-GFP* and *PTEN* siRNA were simultaneously transfected in half side of opaca area. (**C′**) The magnification image from dotted line square in panel C. (**D**) The merge image of panels B,C. (**D′**) The magnification image from dotted line square in panel D. (**E**) The blood islands density chart for the incidence of phenotype above. Abbreviation: bi, blood islands. Scale bars: 1 mm in A; 300 µm in B,C,D; 100 µm in A′,B′,C′,D′.

The mesoderm cells that migrated laterally–caudally from primitive streak will differentiate into blood islands. Hence, we examined the embryo unilaterally co-transfected with *PTEN*-siRNA and *GFP* to establish the effects of silencing *PTEN* on blood islands development (Fig. 4A,B,D,E). The *PTEN* silencing was confirmed by *in situ* hybridization (Fig. 4B) and immunocytochemistry (Fig. 4C). The early (Fig. 4B) and late stages (Fig. 4D) of blood islands formation in the area opaca following *PTEN* knockdown were examined. We found the blood islands were abnormally formed at the *PTEN*-siRNA transfected side (Fig. 4B,D) compared with the control side. To further verify the effect of endogenous PTEN on blood islands formation, we employed *VE-Cadherin in situ* hybridization as blood islands marker following downregulating PTEN with *PTEN* siRNA and *GFP* co-transfection (Fig. 4F,G). The result show that *PTEN* siRNA side blood islands density (Fig. 4F″) decreased obviously in comparison to control side (Fig. 4F′). This implies that the blood islands could not be properly created without *PTEN* participation. At the same time, we can rescue this result by co-transfection *PTEN* siRNA and *wt PTEN-GFP* (Fig. 5). We found the blood islands were equally formed at the *PTEN* siRNA and *wt PTEN-GFP* co-transfected side (Fig. 5B′,C′,D′) compared with the control side (Fig. 5A′). This implies that the blood islands induced by knockdown of *PTEN* previously could be rescued when co-transfection of *wt PTEN-GFP*.

### Overexpression of PTEN impairs mesodermal cell contribution to blood islands

**Fig. 6.**
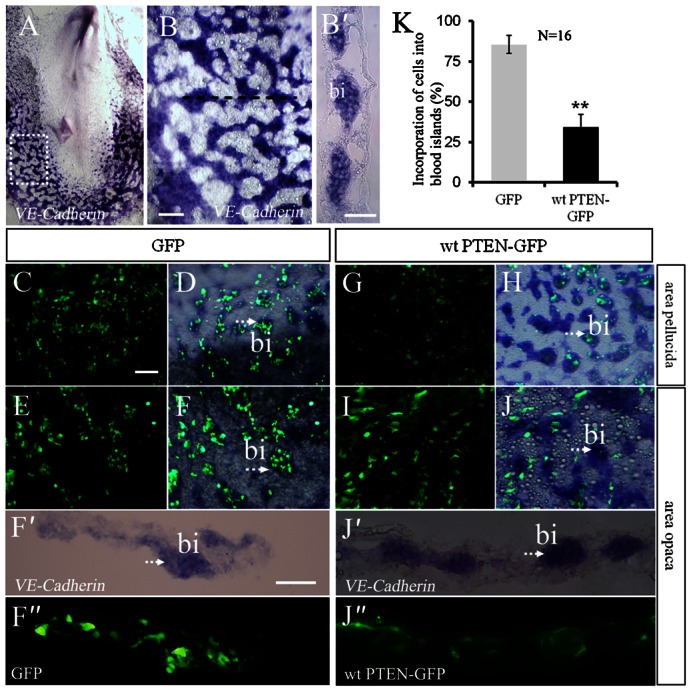
Overexpression of PTEN obstructs the incorporation of mesodermal cells into the blood islands. (**A–B′**) *In situ* hybridization revealing the *VE-Cadherin* expression pattern during chick gastrulation. (A) *VE-Cadherin* is mainly expressed in the head, neural tube and blood islands. (B) Higher magnification and (B′) transverse section of panel A. (**C**) Showing the presence of GFP^+^ cells and (**D**) merge (GFP + *VE-Cadherin*) in the area pellucida. *VE-Cadherin in situ* hybridization was performed 30 hours after GFP^+^ tissues were transplantation. (C,D) Showing almost all of the GFP^+^ cells were incorporated into *VE-Cadherin^+^* blood islands. (**E**) Showing the presence of GFP^+^ cells and (**F**) merge (GFP + *VE-Cadherin*) in the area opaca 30 hours after GFP^+^ tissues were transplantation. Again, most of the GFP^+^ cells were incorporated into *VE-Cadherin^+^* blood islands. (**F′**,**F″**) Transverse sections of panel F showing GFP^+^ cells were uniformly distributed in *VE-Cadherin^+^* blood islands. (**G**,**H**,**I**,**J**) *In situ* hybridization for *VE-Cadherin* was performed 30 hours after wt PTEN-GFP*^+^* tissue transplantation. The results showed very few wt PTEN-GFP*^+^* cells have incorporated into the blood islands as compare with GFP*^+^* cells. (**J′**,**J″**) Transverse sections of panel J showing wt PTEN-GFP*^+^* cells were distributed in the peripherally of blood islands. (**K**) Chart revealing a significant difference in the distribution and incorporation of GFP*^+^* and wt PTEN-GFP*^+^* cells in the blood islands. Abbreviation: bi, blood islands. Scale bars: 500 µm in A; 100 µm in B,C–F,G–J; 50 µm in B′,F′,F″,J′,J″.

*VE-Cadherin* is an adhesion molecule highly expressed by cells in the blood islands and by endothelial cells of blood vessels that later formed. Using *in situ* hybridization, we showed that *VE-Cadherin* was initially expressed by cells in the blood islands of area opaca (Fig. 6A,B,B′) and intra-embryonic area pellucida (Fig. 6A,B,B′). Transplantation of GFP^+^ mesodermal cells indicated that almost all of these cells give rise to blood islands in the area opaca and pellucida (Fig. 6C–F,F″). There was no change in *VE-Cadherin* expression in the blood islands of area opaca or pellucida following the transplantation of wt PTEN-GFP^+^ graft (Fig. 6G–J). However, less than half of the wt PTEN-GFP^+^ cells failed to integrate into the blood islands (Fig. 6G–J,J″). Specifically, there were significantly fewer wt PTEN-GFP^+^ cells in the area opaca (Fig. 6J) than the area pellucida (Fig. 6H). This suggests that proper *PTEN* expression is required for the migrant mesodermal cells to be recruited into the blood islands. Another interesting phenomenon that we identified was the presence of numerous wt PTEN-GFP^+^ cells distributed at the periphery of blood islands. Normally, the peripheral cells of the blood islands differentiate into endothelial cells of blood vessels during development.

**Fig. 7.**
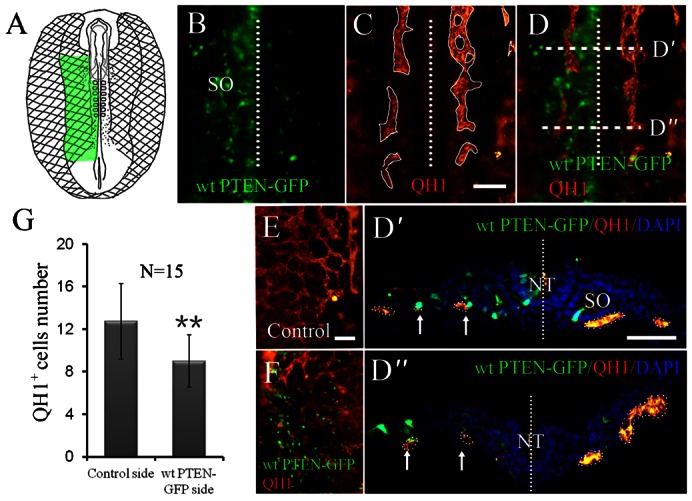
PTEN overexpression hinders intra-embryonic vasculogenesis in quail. (**A**) Schematic drawing showing vasculogenesis (reticular) in both extra- and intra-embryo during gastrulation. (**B**) Wt PTEN-GFP was overexpressed on half side (left) of the early quail intra-embryo (as indicated by green in panel A). (**C**) Immunostaining for QH1 (a quail endothelial cell marker) following wt PTEN-GFP overexpression. (**D**) Merge images of panels B,C showing that the formation of primary vascular plexus was reduced by wt PTEN-GFP overexpression side (left). (**D′**,**D″**) Transverse sections of panel D showing less QH1 expressions on the wt PTEN-GFP overexpressed side (dotted lines indicate the mid-line of the embryo). (**E**,**F**) QH1 expression on the control (E) and wt PTEN-GFP overexpressed sides (F). The two representative images were merged and again demonstrated the primary vascular plexus was reduced following wt PTEN-GFP overexpression. (**G**) The graph illustrates that the incidence QH1^+^ cells number of the control and wt PTEN-GFP side. Abbreviations: SO, somite; NT, neural tube. **P<0.01 vs control side. Scale bars: 100 µm in B–D,E,F; 50 µm in D′,D″.

To further understand the role of *PTEN* in vasculogenesis, we investigated intra-embryonic vasculogenesis using the same method as we did for extra-embryonic vasculogenesis. We overexpressed wt PTEN-GFP in the area pellucida of quail embryos since intra-embryonic vasculogenesis arise there (as schematically shown in Fig. 7A). QH1 (a specific marker for quail endothelial cells) was used to visualize the intra-embryonic primary vascular plexus following wt PTEN-GFP overexpression on one side of early quail embryo. We found that overexpressing wt PTEN-GFP dramatically inhibited the development of vascular plexus when compared with the control side (Fig. 7B–D). We compared the wt PTEN-GFP transfected regions (Fig. 7E,F) with transverse sections of the embryos (Fig. 7D′,D″) to verify the observation. The findings suggest that overexpression of *PTEN* interfere with intra-embryonic vasculogenesis.

### Lipid phosphatase activity is crucial for PTEN participation in vasculogenesis

**Fig. 8.**
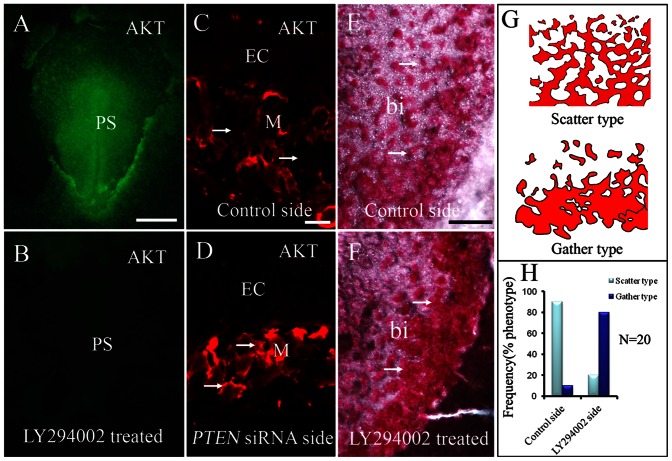
PTEN lipid phosphatase activity and vasculogenesis. (**A**) Immunostaining for AKT in whole-mount HH4 chick embryo. AKT is chiefly expressed in the primitive streak and lateral mesoderm. (**B**) The AKT^+^ staining was completely eliminated when HH4 chick embryo exposed to 4 µM LY294002. (**C**,**D**) Immunostaining for AKT following transfection with *PTEN-*siRNA at half side of the embryo (D) while the contralateral side served as the control (C). The staining revealed that AKT was augmented after *PTEN* expression was silenced (arrows). (**E**,**F**) Area opaca stained with carmine dye, the embryo was unilaterally exposed to 4 µM LY294002 (F) while the contralateral side was the control (E). In the presence of LY294002, the blood islands were found abnormally aggregated in the area opaca compared to control – as illustrated in the schematic drawing (**G**). (**H**) Showing 80% of the LY294002 treated embryos produced abnormal and aggregated blood islands. Abbreviations: PS, primitive streak; EC, ectoderm; M, mesoderm; bi, blood islands. Scale bars: 1 mm in A,B; 20 µm in C,D; 300 µm in E,F.

PTEN protein can act as a phosphatase to dephosphorylate phospho-tyrosine, serine and threonine and also dephosphorylate PtdIns(3,4)P2 and PtdIns(3,4,5)P3. Wt PTEN contain its PtdIns(3,4,5)P3 lipid phosphatase activity, suppressing phosphoinositide 3-kinase (PI3K)-dependent signaling pathways. Wt PTEN also possesses a protein phosphatase activity. PTEN mutant (PTEN G129E) has only protein phosphatase activity. Another PTEN mutant (PTEN C124S) has lack two phosphatase activity (Leslie et al., 2007). The question we want to ask is which phosphatase is predominant in regulating embryonic vasculogenesis. To address this question, we treated HH3 stage chick embryos with the LY294002 inhibitor (Vlahos et al., 1994) because it can specifically suppress PI3K activity. Since AKT is an important component in PI3K signaling, we used it as a marker for PI3K-AKT signaling activity. We determined that P-AKT was highly expressed in early HH3 chick embryos (Fig. 8A), and that AKT expression was abolished following exposure to 4 µM of LY294002 (Fig. 8B). However, the results only indicate that PI3K-AKT signaling is associated with activities in the early chick embryo. There is no evidence to support the idea of a crosstalk between the phosphatase of *PTEN* gene and chick embryonic vasculogenesis. To establish whether there was this link we immunofluorescent stained early chick embryos with P-AKT antibody following *PTEN* silencing. The results showed that P-AKT expression was dramatically increased in the *PTEN* silenced mesodermal cells (Fig. 8D) compared with the untransfected side (Fig. 8C). For the LY294002 treated chick embryos, it lead to morphologically abnormal blood islands being formed (Fig. 8F) compared with blood islands formed on the control side (Fig. 8E). The abnormal blood islands that formed were highly aggregated and lost their normal morphology as schematically illustrated in Fig. 8G. This abnormality was evident in approximately 80% of the total LY294002 treated embryos (Fig. 8H). These results suggest that the *PTEN* lipid phosphatase activity plays a predominant role in *PTEN*-mediated vasculogenesis.

In order to exclude the possibility that protein phosphatase activity of PTEN, we transfected either C124S PTEN-GFP (both lipid and protein phosphatase mutated) or G129E PTEN-GFP (lipid phosphatase mutated) unilaterally in HH3 early chick embryos as previously described (supplementary material Fig. S3). The results demonstrated that neither C124S PTEN-GFP nor G129E PTEN-GFP transfection have effect on blood islands formation as shown hereunder. Blood islands density and morphology following the transfection of the either C124S PTEN-GFP or G129E PTEN-GFP have not alternated in comparison to control side (supplementary material Fig. S3C,D,G,H), suggesting that the lipid phosphatase of PTEN play more principal role on regulating blood islands formation.

### Abnormal blood islands formation induced by *PTEN* overexpression do not involve VEGF signaling

**Fig. 9.**
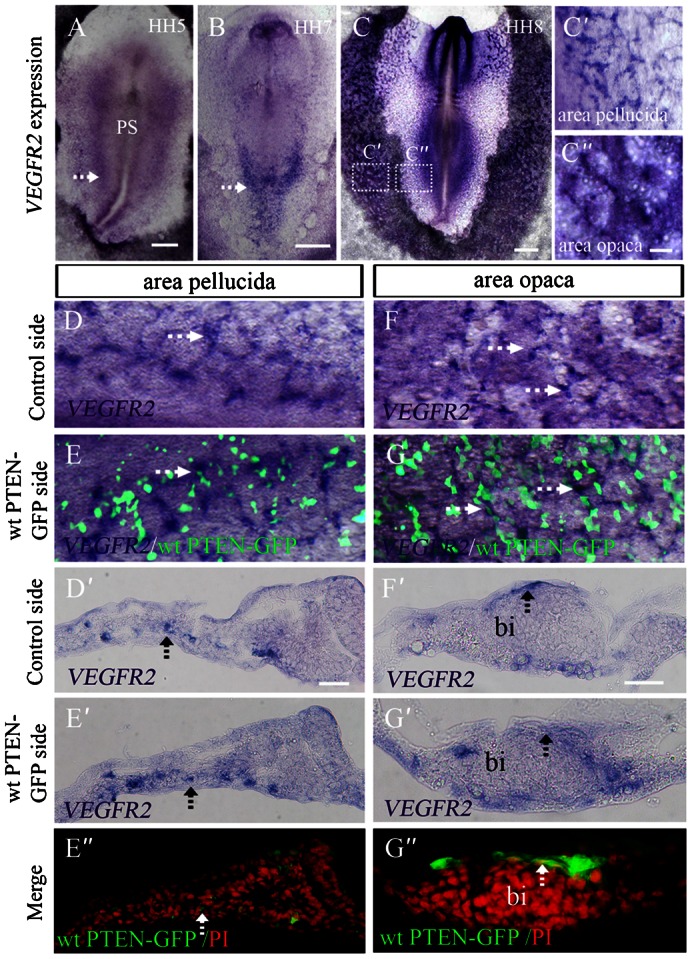
Abnormal blood islands produced by PTEN overexpression are not associated with VEGF signaling. (**A–C″**) *In situ* hybridization showing *VEGFR2* is expressed in the lateral mesoderm adjacent to the primitive streak in area pellucida in HH5 and HH7 chick embryos indicated by white arrowheads (A,B). *VEGFR2* is also expressed in the head folds, intra-embryonic area pellucida and blood islands in the area opaca (C,C′,C″). (**D**,**E**) *VEGFR2* expression in area pellucida was determined by *in situ* hybridization following *wt PTEN-GFP* transfection (E), and control side (D) was indicated by white arrowheads. (**D′**,**E′**,**E″**) The transverse sections were from panels D,E respectively, in which *VEGFR2* expression were not much different between wt PTEN-GFP and control side indicated by dotted black arrowheads in D′,E′. E″ is the merge of wt PTEN-GFP expression and PI staining. (**F**,**G**) *VEGFR2* expression in area opaca was determined by *in situ* hybridization following *wt PTEN-GFP* transfection (G), and control side (F) was indicated by white arrowhead. (**F′**,**G′**,**G″**) The transverse sections (F′,G′) were from panels F,G respectively. *VEGFR2* expression between wt PTEN-GFP transfection and control is resemble as in area opaca as indicated by black arrowheads in F′,G′. The results demonstrated that *VEGFR2* expression was not affected by wt PTEN-GFP overexpression. PI staining was used to plot the blood island. Wt PTEN-GFP*^+^* cells were found localized on the periphery of blood islands (G″). Abbreviations: PS, primitive streak; bi, blood islands. Scale bars: 1 mm in A,B,C; 200 µm in C′,C″,D,E,F,G; 50 µm in D′,E′,E″,F′,G′,G″.

It has been well established that VEGF signaling plays a very important role in embryonic vasculogenesis – as it regulates endothelial cell proliferation and migration (Shibuya and Claesson-Welsh, 2006). Consequently, we investigated whether the abnormal blood islands that formed as a result of *PTEN* overexpression was attributed to disrupted VEGF signaling. We first performed *in situ* hybridization to elucidate where *VEGFR2* (the most important receptor of VEGF ligands in early chick embryo) was expressed in the embryo (Fig. 9A–C). *VEGFR2* was found expressed in the blood islands of extra-embryonic area opaca (Fig. 9C,C″) and intra-embryonic area pellucida (Fig. 9C,C′). We found that wt PTEN-GFP did not alter *VEGFR2* expression in hemangiblasts of blood islands of area pellucid (Fig. 9D,E), and mainly expression in angioblasts of blood islands of area opaca (Fig. 9F,G), which was confirmed by comparing transverse sections of wt PTEN-GFP (Fig. 9E′,G′) and control sides (Fig. 9D′,F′). Most of the wt PTEN-GFP^+^ cells were located in the periphery of blood islands (Fig. 9G″). The results suggest that the malformation of blood islands induced by *PTEN* overexpression was not through perturbed VEGF signaling.

## Discussion

Vasculogenesis is the process where de novo blood vessels are formed from migratory mesodermal cells. During gastrulation, the epiblast cells undergo EMT in the caudal region of the primitive streak and emigrate laterally and caudally to the extra-embryonic area opaca (i.e. the yolk-sac, as illustrated in Fig. 1). At the area opaca, the mesodermal cells give rise to the blood islands. *PTEN* is robustly expressed in the primitive streak and the blood islands in the area opaca. This expression pattern is spatiotemporally correlated with the morphogenetic processes that occur during vasculogenesis and suggests that *PTEN* might be involved. EMT is a gene-modulated conversion process where epithelial cells convert into mesenchymal cells during both embryogenesis and tumorigenesis. Kim et al. reported that *PTEN* was essential for maintaining the cellular adhesion between retinal pigment epithelial cells (Kim et al., 2008). In *PTEN* knockout mice, these epithelial cells undergo EMT rapidly and migrate out quickly due to decreased cell adhesiveness. Presently, we have also obtained similar phenotype when we overexpressed or silenced *PTEN* in the early chick embryo. We discovered that when *PTEN* was overexpressed during gastrulation, it resulted in fewer emigrating mesodermal cells owing to the disruption of the EMT process. Likewise, silencing *PTEN* also obstructed the formation of the mesoderm germ layer and the migration of mesodermal cells to the area opaca. This was evident from examining the thickness of the mesoderm layer which was significantly thinner in the *PTEN*-silenced side than the contralateral control side. There was also fewer blood islands formed in the area opaca. There are many possible causes for the production of these ambivalent phenotypes. One possibility is that *PTEN* is a multifunctional gene that plays many diverse roles which are dependent on the context, such as the developmental stage of the embryo or the different sites/environments that the hemangioblasts encountered during their migration. For instance, *PTEN* could be exerting its effect during (1) EMT, (2) lateral–caudal emigration, (3) cell aggregation at the blood islands in the area opaca, and (4) differentiation of the hematopoietic and endothelial linages. This hypothesis is supported by our results where we elucidated the migration and development fate of wt PTEN-GFP^+^ mesodermal cells (derived from transplanted primitive streak tissues transfected with *wt PTEN-GFP*).

Presently, we have used *VE-Cadherin* as a marker to follow the development of the blood islands. We found that when posterior primitive streak tissue were transfected with *GFP* or *wt PTEN-GFP* and then transplanted into host embryos, no abnormal *VE-Cadherin*-labeled blood islands were formed. The reason for this is because there were far fewer wt PTEN-GFP^+^ mesodermal cells present in the total makeup of the migrating mesodermal cell population. Therefore, the wt PTEN-GFP^+^ cells had a minimal influence on directing how the blood islands were formed. Interestingly, we also noticed that the wt PTEN-GFP^+^ mesodermal cells did not incorporate themselves into the blood islands but distributed themselves on the peripherally of the islands. In fact, they appeared to avoid the blood islands which contrast with the GFP^+^ mesodermal cells which contributed almost exclusively to the blood islands (compare Fig. 6F with Fig. 6J). This suggests that an inappropriate level of *PTEN* in migrating mesodermal cells interfered with their normal function and affected their ability to participate in the formation of blood islands.

Blood islands originate from both intra- and extra-embryo, which would eventually develop into blood vessels in both of these regions. However intra-embryonic blood islands differ distinctly from extra-embryonic blood islands in one respect and that is their inability to generate blood cells, i.e. intra-embryonic hemangioblasts can only produce endothelial cells rather than hematopoietic cells (Godin and Cumano, 2005). In this context, this may perhaps explain why elevated *PTEN* expression disturbed the incorporation of mesodermal cells into the blood islands, which merely appeared in extra-embryo rather than in intra-embryo. The different phenotypes generated in our study also indicate that there is a different mechanism involved in extra- and intra-embryonic vasculogenesis. Furthermore, the circulating cells derived from the blood islands might be able to give rise to new embryonic blood vessels (LaRue et al., 2003). In our study, we noticed that when *PTEN* was overexpressed the hemangioblast cells were diverted to the presumptive endothelial cell linage (Fig. 6J′–J″), which suggests that *PTEN* normally play an important role in regulating the differentiation of hemangioblasts into hematopoietic and endothelial cells in the embryo during vasculogenesis.

*PTEN* belongs to a superfamily of protein tyrosine phosphatase that simultaneously possess robust phosphatase activity against lipids and proteins (Leslie et al., 2009). Presently, we have investigated the role of *PTEN* and cell migration in the context of protein phosphatase activity. Raftopoulou et al. reported that cell migration was inhibited following microinjection of the C2 domain of *PTEN* into glioblastoma cells (Raftopoulou et al., 2004). We have also reported similar phenotype by demonstrating that the protein phosphatase of *PTEN* modulated in the EMT process of chick anterior primitive streak during gastrulation (Leslie et al., 2007). However, in our scenario, we discovered that the principal function of *PTEN* lipid phosphatase was to regulate cell migration in the caudal embryo. We have shown that PI3K-AKT signaling was very active during chick gastrulation and that silencing *PTEN* expression in turn reduces AKT expression. This implies that *PTEN* dephosphorylates PtdInsP3 through its lipid phosphatase. Furthermore, when PI3K signaling was inhibited with LY294002 inhibitor, it resulted in the primary vascular plexus being formed as an aggregated mass of blood islands in the yolk-sac. These findings strongly suggest that PTEN exerted its effect on vasculogenesis primarily through *PTEN* lipid phosphatase activity.

**Fig. 10.**
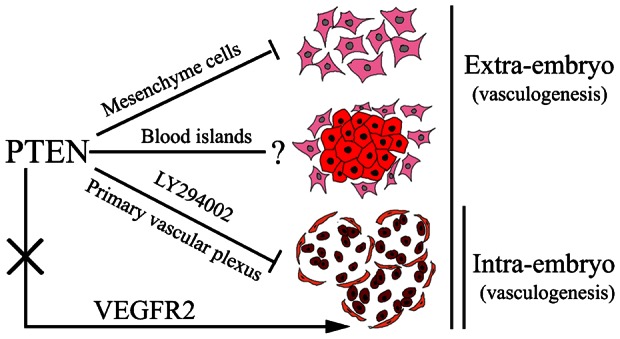
A proposed model depicting the role of PTEN as a participant in embryonic vasculogenesis. In extra-embryo vasculogenesis, PTEN could be exerting its effect during (1) EMT and lateral–caudal mesoderm cell emigration, (2) mesoderm cell aggregation at the blood islands in the area opaca, differentiation of the hematopoietic and endothelial linages, and (3) morphogenesis of blood islands modulated through PTEN/PI3K pathway. In intra-embryo vasculogenesis, PTEN mainly inhibit primary vascular plexus in area pellucida. *PTEN* did not crosstalk with the VEGF signaling pathway in early embryo vasculogenesis.

Eichmann et al. reported that VEGF was indispensible for vasculogenesis in the chick (Eichmann et al., 2002). We have demonstrated that *VEGFR2* was expressed at all stages of vasculogenesis. Therefore, we investigated whether VEGF signaling was involved in *PTEN*-modulated vasculogenesis. We established that *VEGFR2* expression in the area opaca and blood islands were normal and unaffected by *PTEN* overexpression. We have already shown that wt PTEN-GFP^+^ mesodermal cells mainly distributed themselves at peripherally of the blood islands. This indicates that *PTEN* is not relevant to VEGF signaling. We have correlated all of our current findings in a drawing (Fig. 10) to illustrate our proposed model on the role of *PTEN* in embryonic vasculogenesis. Firstly, the EMT process for generating mesoderm cells could be the first target of *PTEN*. Next, *PTEN* plays an indispensable role in regulating mesodermal cell migration and incorporation into blood islands. Finally, *PTEN* is able to direct hemangioblasts in the blood islands to differentiate into angioblasts.

In summary, our results clearly demonstrate an essential multifunctional role for *PTEN* in the modulation of vasculogenesis in the developing chick embryo. Our findings are also comparable to results already reported for higher vertebrates (Godin and Cumano, 2005). Furthermore, the cellular and molecular mechanisms that we have reported were involved in embryonic vasculogenesis may provide new insight into the mechanism of tumor vasculogenesis.

## Supplementary Material

Supplementary Material
