# Supplementary material for: PTEN is involved in modulation of vasculogenesis in early chick embryos
Source: Biol Open. 2013 May 9;2(6):587–95. doi: 10.1242/bio.20133988 (PMC3683161; doi:10.1242/bio.20133988)
Supplement: Supplementary Material [file supp_2_6_587__index.html]

PTEN is involved in modulation of vasculogenesis in early chick embryos — PTEN is involved in modulation of vasculogenesis in early chick embryos — Supplementary Material 

# PTEN is involved in modulation of vasculogenesis in early chick embryos

## bio.20133988 Supplementary Material

**Files in this Data Supplement:**

- Supplementary Material - Yan Li et al. doi: 10.1242/bio.20133988
