# Supplementary material for: PTEN is involved in modulation of vasculogenesis in early chick embryos
Source: Biol Open. 2013 May 9;2(6):587–95. doi: 10.1242/bio.20133988 (PMC3683161; doi:10.1242/bio.20133988)
Supplement: Supplementary Material [file supp_bio.20133988_bio.20133988-s1.pdf]

Biology Open

Supplementary Material

Yan Li et al. doi: 10.1242/bio.20133988

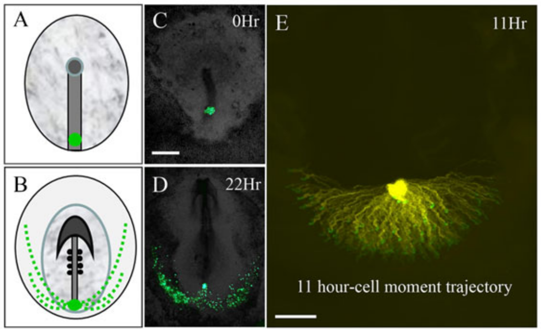

Fig. S1. The trajectory of migrating hemangioblasts from the posterior primitive streak to blood islands. (**A**,**B**) Schematic drawings illustrating the transplantation GFP+ primitive tissue into host embryos (A) 0 hour to (B) 22 hour. Green dot in panel A stands for the graft site in host embryo. The dotted green lines in panel B illustrate the trajectory of the migrating hemangioblasts during development. (**C**) Appearance of the host embryo immediately after grafting. (**D**) The host embryo viewed 22 hour after grafting. (**E**) Illustrating the trajectory of migrating hemangioblast cells 11 hour after transplantation. The large yellow dot marks the graft site in host embryo, while the yellow lines illustrate the trajectory. Scale bars: 1 mm.

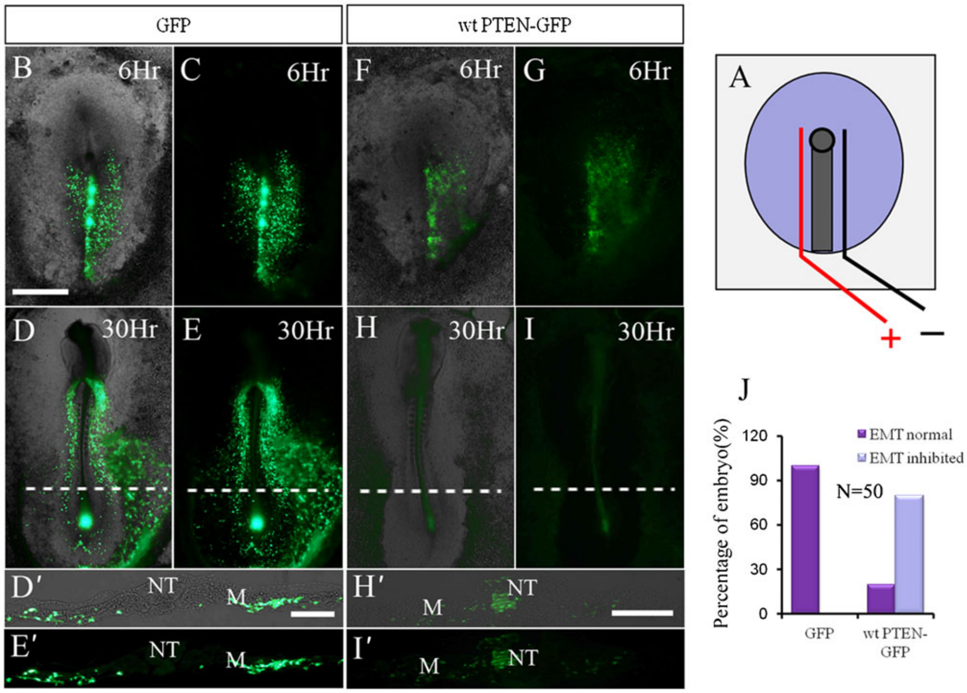

Fig. S2. Overexpression of PTEN disrupts EMT in chick gastrulation. (**A**) Schematic drawings illustrating the transfection of primitive streak stage embryos with *GFP* or *wt PTEN-GFP*. (**B**,**C**) Merge images (bright-field and fluorescence) (B) and fluorescence image (C) of embryos at 6 hours after transfected *GFP*. (**D**,**E**) Merge (D) and fluorescence images (E) of embryos *GFP* transfected after a 30-hour incubation. (**D′**,**E′**) Transverse sections of panels D,E (level indicated by dotted lines) show GFP+ cells were mainly distributed in the mesoderm layer. (**F**,**G**) Merge (F) and fluorescence image (G) of embryos 6 hours after *wt PTEN-GFP* transfected showing few mesodermal cells have migrated out of the primary streak. This was opposite to what was found from embryos transfected with *GFP* alone (B,C). (**H**,**I**) A representative embryo 30 hours after being transfected with a *wt PTEN-GFP*. Almost no GFP+ cells have migrated and become located in the area opaca – suggesting that wt PTEN-GFP overexpression inhibited epithelial–mesenchymal transition. (**H′**,**I′**) Dotted lines indicate transverse sections made from panels H,I respectively. These sections revealed an accumulation of GFP+ cells present in the midline of the embryo. (**J**) The rate of EMT inhibition was 80% for the total number of embryos transfected with *wt PTEN-GFP*. Abbreviations: M, mesoderm; NT, neural tube; EMT, epithelial–mesenchymal transition. Scale bars: 500 µm in B–D,E,F–H,I; 200 µm in D′,E′,H′,I′.

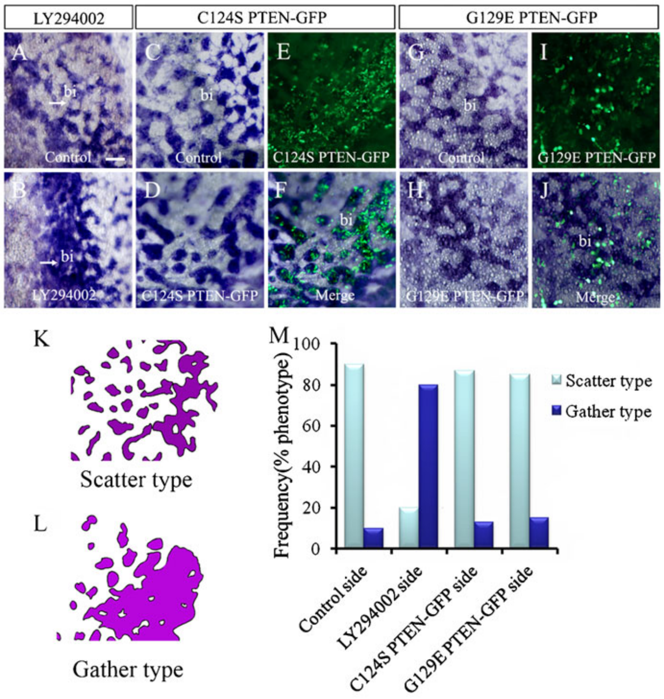

Fig. S3. The activity of lipid phosphatase of PTEN implements its major function on vasculogenesis. (**A**,**B**) The both *VE-Cadherin* in situ hybridization images are from bilateral area opaca of the embryo, in which was exposed to 4 µM LY294002 at unilateral embryo (B) while left another side (A) as control. (**C**,**D**) Both the *VE-Cadherin* in situ hybridization images are from the corresponding sites of bilateral area opaca of the embryo, which was transfected by overexpression C124S PTEN-GFP at unilateral embryo (D) while left another side (C) as control. (**E**) C124S PTEN-GFP transfected on opaca area. (**F**) The merge image of panels D,E. (**G**,**H**) *VE-Cadherin* in situ hybridization images are from the corresponding sites of bilateral area opaca of the embryo, which was transfected by overexpression G129E PTEN-GFP at unilateral embryo (H) while left another side (G) as control. (**I**) G129E PTEN-GFP was transfected on opaca area. (**J**) The merge image of panels H,I. The formation of blood islands were abnormally aggregated in unilateral area opaca (B) compared to control (A) as shown in schematically drawing (**K**,**L**), which the incidence of the phenotype is 80% in total LY294002 treated embryos (**M**). Overexpressing C124S PTEN and G129E PTEN did not change blood islands formation in comparison to control side (M). Abbreviation: bi, blood islands. Scale bar: 100 µm in A–J.

Biology Open
